# Tailor-made natural and synthetic grafts for precise urethral reconstruction

**DOI:** 10.1186/s12951-022-01599-z

**Published:** 2022-08-31

**Authors:** Qinyuan Tan, Hanxiang Le, Chao Tang, Ming Zhang, Weijie Yang, Yazhao Hong, Xiaoqing Wang

**Affiliations:** 1 Department of Urology, The First Hospital of Jilin University, 1 Xinmin Street, Changchun, 130061 People’s Republic Of China; 2 Department of Orthopedics, The Second Hospital of Jilin University, 218 Ziqiang Street, Changchun, 130041 People’s Republic Of China; 3 Department of Pediatric Surgery, The First Affiliated Hospital of Nanjing Medical University, 300 Guangzhou Street, Nanjing, 210029 People’s Republic Of China

**Keywords:** Natural graft, Synthetic graft, Biomimetic structure, Tissue engineering, Precise urethral reconstruction

## Abstract

Injuries to the urethra can be caused by malformations, trauma, inflammation, or carcinoma, and reconstruction of the injured urethra is still a significant challenge in clinical urology. Implanting grafts for urethroplasty and end-to-end anastomosis are typical clinical interventions for urethral injury. However, complications and high recurrence rates remain unsatisfactory. To address this, urethral tissue engineering provides a promising modality for urethral repair. Additionally, developing tailor-made biomimetic natural and synthetic grafts is of great significance for urethral reconstruction. In this work, tailor-made biomimetic natural and synthetic grafts are divided into scaffold-free and scaffolded grafts according to their structures, and the influence of different graft structures on urethral reconstruction is discussed. In addition, future development and potential clinical application strategies of future urethral reconstruction grafts are predicted.

## Background

The lower urinary tract comprises the bladder, urethra, and urethral sphincter, and it is mainly responsible for storing and excreting urine [1]. The bladder and urethra are primarily composed of two layers of cells, the urothelium and the smooth muscle cell layers [2]. The urothelium cell layer can prevent urine erosion and inhibit toxin absorption, while the smooth muscle cell layer is responsible for the elasticity and flexibility of the lower urinary tract [3, 4]. There are also widely distributed small blood vessels in the tissues, which nourish the surrounding tissues.

There are many causes of urethral injuries, including malformations, inflammation, trauma, and carcinoma. These injuries frequently lead to urethral stricture, and the stricture can result in bladder calculi, fistulas, sepsis, and ultimately renal failure [5]. The pathology of urethral stricture is characterized by changes in the extracellular matrix of urethral tissue [6]. The normal connective tissue is replaced by dense fibers distributed by fibroblasts in stricture tissue. Moreover, the ratio of type III to type I collagen is also decreased [7]. Reconstruction of the injured urethra is still a challenge for urological surgeons. Different repair strategies are used according to the length, location, and cause of the damage [8]. To date, urethral injuries involving a shallow corpus cavernosum can be cured by urethroplasty and end-to-end anastomosis, but the recurrence rate is high [9]. There is no way to repair long urethral injuries entirely relying on the migration and proliferation of surrounding autologous tissue cells. Therefore, it is necessary to construct a suitable urethral graft for urethral repair. Most clinical treatments for urethral injuries use autologous tissue substitutes, such as skin flaps or buccal mucosa (BM) [10]. However, studies have shown that the acquisition and transplantation of skin flaps is more technically complicated, and patients do not prefer this option [11]. Donor tissues such as BM or free skin grafts are not always available [12]. These interventions also have limitations, such as donor site injuries, fistulas, and a high recurrence rate in the repaired area [13]. At present, the clinical application of urethral tissue engineering is mainly based on porcine small intestinal submucosa (SIS) patch(SURGISIS®, Cook Medical). However, as an outdated material, SIS patch has poor cell penetration and growth [14], and has high recurrence rate (24%) in clinical trials [15].

The limitations of clinical treatment for urethral injuries have promoted the development of urethral tissue engineering. The tissue-engineered urethra mimics the native microenvironment of the urethra by combining cells with inducing factors and scaffolds [16]. The urethral scaffold is suitable for cell growth and mechanical support and acts as a bridge for cell migration and aggregation. During the entire urethral reconstruction process, the structure of the graft plays a vital role, whether from the micro- or macroscopic perspective. The importance of graft structure in urethral reconstruction has received wide attention. A suitable graft structure can promote the regeneration of tissues to approach the natural urethra, thereby speeding up the repair process and increasing the success rate.

This review discusses the impact of graft structure on the repair of urethral defects from the micro- and macroscopic perspectives of the urethral graft structure. We further discuss the advantages of three-dimensional (3D) bioprinting and new hydrogels in urethral reconstruction and possible future development directions. In addition, we review the potential clinical application strategies of urethral tissue-engineered grafts. This work provides a comprehensive review of urethral tissue engineering grafts with different structures for urethral reconstruction.

## Biomimetic grafts for urethral reconstruction

**Fig. 1:**
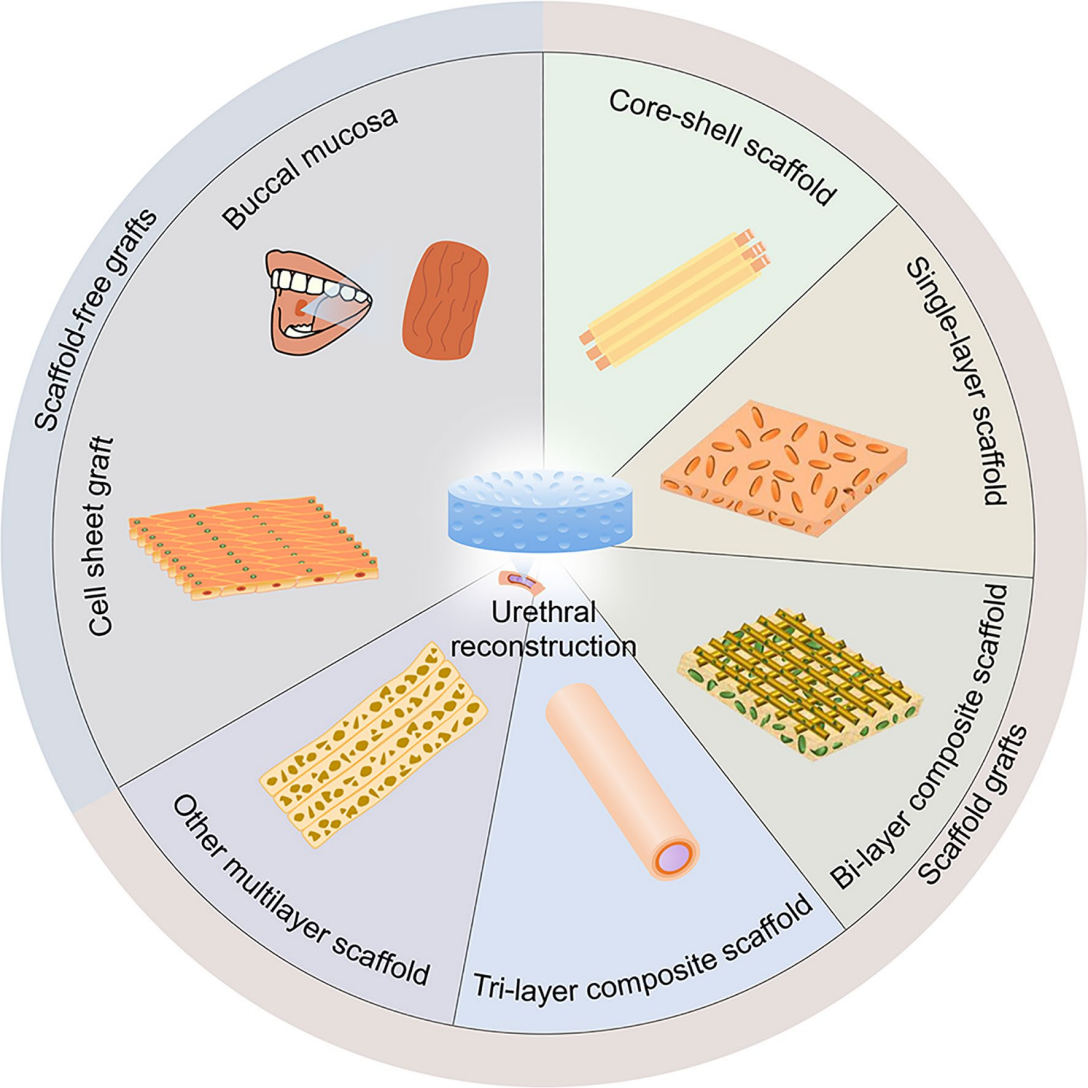
Schematic illustration of tailor-made natural and synthetic grafts for precise urethral reconstruction

Making the regenerated urethral tissue similar in structure and function to the natural urethra is the ultimate goal of urethral tissue engineering. Scholars have made many attempts to select scaffold materials and cells to achieve this goal. Nevertheless, the macro- and micro-structure of the scaffold also plays a crucial role in urethral regeneration [17–19]. In this review, we divide urethral grafts into two types according to their structures: scaffold-free and scaffolded grafts. Additionally, we define a scaffold as an artificial graft with 3D support, cell-bearing function, and considerable strength. Figure 1 provides a brief systematic summary of this review.

## Scaffold-free grafts

Scaffold-free grafts include flaps, patches, and cell sheet grafts. Grafts without scaffold structures are commonly used for urethral reconstruction in the clinic. The use of BM and flaps to repair urethral defects is the clinical gold standard treatment option [20]. Scaffold-free grafts usually use autologous tissues and cells. The urethral repair environment is closer to natural urethral tissue and has low immunogenicity compared with scaffolded grafts. Table 1 summarizes the scaffold-free grafts used in urethral reconstruction.

**Table 1:** Overview of studies describing the use of scaffold-free grafts in urethral reconstruction

Scaffold-free grafts	Biomaterials	Clinical/preclinical experiment	Animals for modeling	Average repair length	Follow-up/study period	In vivo/In vitro	Results	References
Flaps and patches	Dorsal penile flap	Clinical	NA	5 cm	3years	In vivo	Overall success rate is 88%; restore urethra function; provide cosmetic effects	[21]
	Oral mucosal grafts	Clinical	NA	3–5 cm	At least 1year	In vivo	overall success rate is 85%; reduced operation time	[24]
	BM	Preclinical	Rabbit	1.5 cm	3months	In vivo	induce angiogenesis; repair urethra tissue effectively	[27]
	Skeletal muscle	Preclinical	Rabbit	0.5 cm	12 weeks	In vivo	provide a large amount of angiogenic cytokines highly vascularized porosity flexibility;	[32]
	Induced microtissues	Preclinical	NA	NA	NA	In vivo	functional layering; single continuous urothelium	[34]
Cell sheet grafts	Oral epithelial cell and muscle cells	Preclinical	Canine	2 cm	12 weeks	In vivo	stratified urothelium intact muscle layer wide caliber	[36]
	Fibroblasts, ECs and UCs	Preclinical	NA	NA	NA	In vivo	promote the formation of blood vessel reduce necrosis;	[37]
	Bidirectionally induced ADSCs	Preclinical	Rabbit	2 cm	6months	In vivo	stable multilayer epithelial cell layer; significant vascularization; Visible smooth muscle layer	[39]
	Oral mucosal epithelial cells, oral fibroblasts and ADSCs	Preclinical	Canine	2 cm	3 months	In vivo	regenerated tissue similar to natural urethra	[40]

*NA* not available

### Flaps and patches

The penis skin flap was once considered an excellent urethral substitute because it is easy to harvest, hairless, and compatible in humid environments. Additionally, it is a flexible tissue with an abundant blood supply suitable for reconstructing long and complex urethral injuries. Hmida et al. [21] applied dorsal penile flap urethroplasty to treat 77 patients with urethral stricture. After surgery for 1 year, 5% of patients developed recurrent stenosis. The penis skin flaps restore urethral function and provide good cosmetic effects in the repaired area. Some studies have shown that BM and penile skin flaps have the same success rate [22, 23]. Barbagli et al. [24] used oral mucosal grafts to perform penile urethroplasty in 14 patients, and the postoperative follow-up time was more than 12 months. During the operation, Glubran 2 (GEM, Viareggio, Italy) was used to assist the oral mucosa in adhering to the urethral plate, stopping bleeding, and shortening the operation time (approximately half an hour). In the end, 12 of the 14 patients were considered successful, and none of the patients responded that it affected sexual function. Although both have high success rates, transplantation of BM grafts may be the preferred option because of the shorter surgical time and more straightforward technique [25, 26]. The main reason for the failure was insufficient blood supply, leading to the recurrence of inflammation and stenosis. To increase the graft’s vascular supply and improve the graft’s survival rate, Guo et al. [27] designed an anterior capsular BM composite graft for urethral reconstruction in rabbits. The formation of vascularized capsules was induced by inserting a tissue expander into the groin. Then, BM grafts were transplanted into capsule tissue with axial blood vessels, and BM-lined flaps were prefabricated. The capsule served as an induced vascular bed for BM-lined flaps, which greatly enhanced the vascularization of the graft. This prefabricated capsule-BM composite flap has an independent vascular distribution and effectively repairs the urethra. However, BM grafts require additional oral surgery and specialized nursing [28]. Therefore, some scholars have proposed that if small (< 3 cm) grafts are needed in urethral reconstruction surgery, “mini-patch” penile skin grafts are an effective alternative to obtaining BM grafts [29]. However, this still did not solve the problem of requiring multiple operations.

The pedicled muscle flap demonstrates good mechanical properties and complete tissue microstructure, and it has been widely used in regenerative medicine [30, 31]. Sun et al. [32] mixed hypoxia-activated human umbilical cord mesenchymal stem cells (hUCMSCs) and rabbit skeletal muscle in the subcutaneous cavity of the ventral rabbit penis for 3 weeks to prepare vascular anterior urethral grafts. The construct was then used as a patch graft to repair rabbit urethral defects. Hypoxic preconditioning of hUCMSCs provided many angiogenic cytokines required for angiogenesis [33]. Patch grafts are highly vascularized with good porosity and flexibility, making them excellent biomaterials for urethral reconstruction. Although progress has been made in the urethral reconstruction of the penis skin flap, BM, and muscle flap, their tissue structure is different from that of the urethra. The additional surgical damage increases the patient’s physical burden. Therefore, grafts may need to simulate the urethral structure to reconstruct the urethral injury. Jin et al. [34] used human adipose-derived stem cells (hADSCs) in vitro to induce grafts with urethral tissue structure. They promoted the differentiation of hADSCs into induced microtissues (ID-MTs). ID-MTs were subcutaneously embedded in nude mice for 1 week and then seeded with human urothelial cells (hUCs) to form tissue-engineered urinary tract patches. Histological analysis showed that ID-MTs could mimic the smooth muscle layer of the natural urethra, and the implanted hUCs could also be observed as a single continuous layer. This shows that the patch constantly develops to conform to the natural urethral structure and is also an alternative to using autologous tissue directly.

### Cell sheet structure grafts

The urethra comprises multiple layers of cells and functional tissues, mainly urothelial and smooth muscle cells (SMCs). According to the structural and cell composition of the urethra, cell sheet technology combines various types of cell sheets to form the biomimetic urethra, which is more intuitive. As early as 2008, Nagele et al. [35] successfully cultured human urothelial cell monolayer cultures to produce multilayer urothelial cell sheets. This makes it possible to prepare biomimetic urethral grafts using cell sheet technology. Mikami et al. [36] collected oral tissues by puncture biopsy and isolated oral epithelial cells and muscle cells. These two types of cells were cultured as muscle and epithelial cell sheets. The sheets were combined to form tubular biomimetic urethras and autografted into canine models of the urethral defect. The neourethra showed stratified urothelium and intact muscularis, and the urethra remained open for 12 weeks postoperatively. Imbeault et al. [37] cultured human fibroblasts for 4 weeks to prepare fibroblast sheets and seeded endothelial cells (ECs) to form a tubular structure. Then, UCs were inoculated into the lumen to reconstruct the urethra in mice. The presence of endothelial cells promoted the early formation of blood vessels in the tubular urethra and reduced the necrosis of transplanted tissue. Fibroblasts can produce a dense ECM, closely mimicking the natural urethral microenvironment. With the help of the functional properties of seeded cells and reasonable spatial distribution, the survival rate of the graft is greatly improved.

**Fig. 2:**
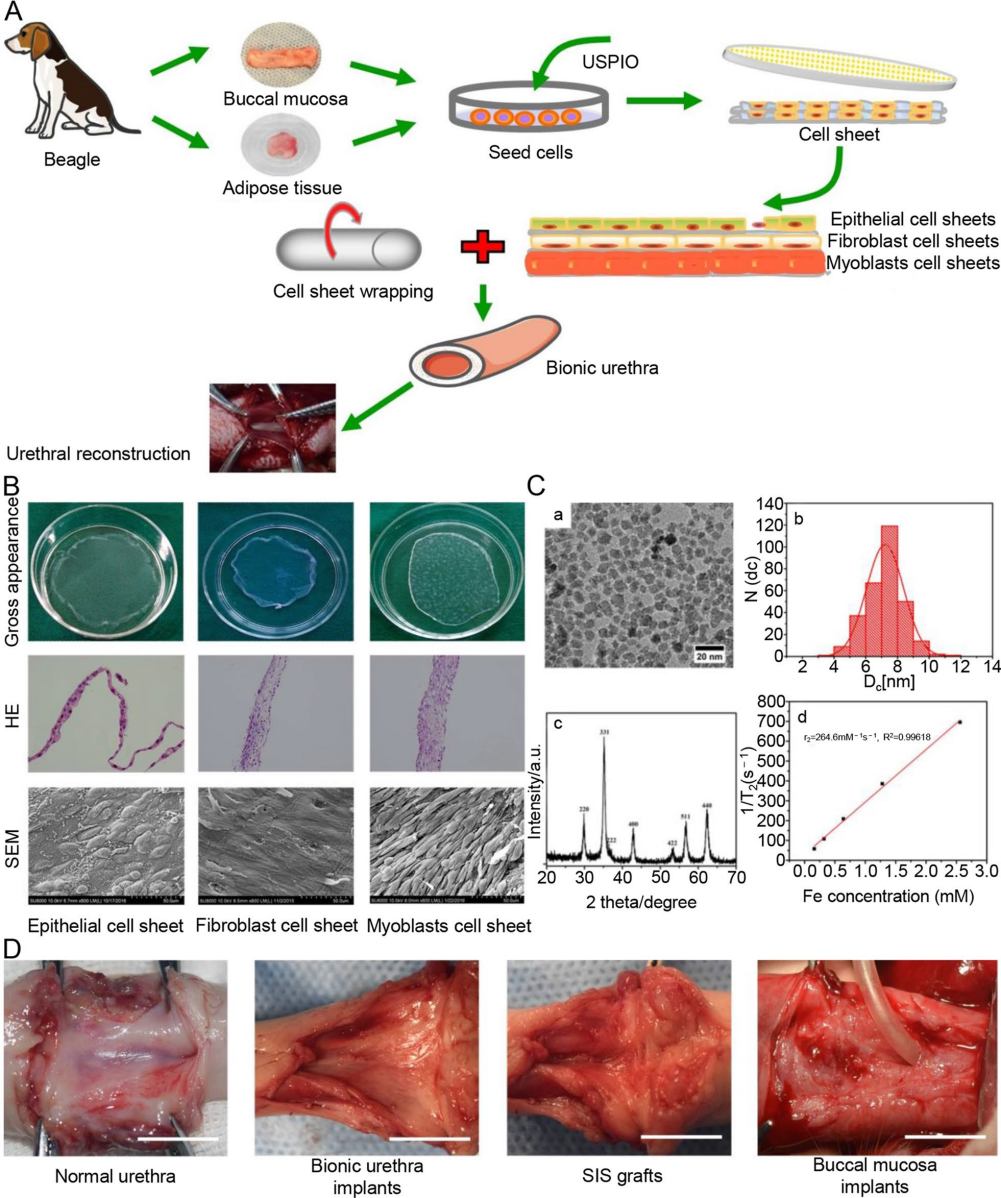
Overview of key components in the study. **A** The scheme diagram of tissue-engineered bionic urethras using cell sheet technology. **B** Cell sheet formation and identification. Cell sheet formation after 21 days of continuous culture. The first line shows photographs of each of the types of cell sheets; the second line show H&E staining results of three cell sheets, which revealed that the cultured oral mucosal epithelial cell sheets were composed of 2–3 layers of cells (left, scale bar: 50 μm), the cultured oral fibroblast cell sheets were composed of 3–4 layers of cells (middle, scale bar: 100 μm), and the cultured myoblast induction of ADSCs sheets were composed of 6–7 layers of cells (right, scale bar: 100 μm); the third line shows the SEM images of the three cell sheets, scale bar: 50 μm. **C** Characterization of USPIO **a** TEM image; **b** the diameter distribution from TEM; **c** The XRD pattern of the synthesized USPIO; **d** The value of T2 relaxation rate of the synthesized USPIO as a function of Fe concentration from MRI of USPIO in tissue-engineered bionic urethra. **D** Macroscopic examination of retrieved urethra at 3 months after full-thickness urethral reconstruction. Similar to normal urethra, no ulcerations, strictures, and fistulas were observed in bionic urethra implants and buccal mucosa implants, but extensive contracture and scarring tissue at the graft site was found in SIS grafts. scale bar: 1.0 cm

Adipose-derived stem cells are widely distributed in the human body, easy to obtain, have strong proliferation ability, can be cultured in vitro to induce differentiation, and have no ethical restrictions; therefore, they are ideal repair materials for urethral reconstruction [38]. Shi et al. [39] performed bidirectional induction of epithelial/smooth muscle cell patches on ADSCs to repair urethral defects in rabbits. Six months later, ADSCs induced the composite membrane bionic urethra to form a complete multilayer urothelium and abundant capillaries without urethral stricture. Zhou et al. [40] used oral mucosal epithelial cell sheets, fibroblast cell sheets, and myoblast-induced ADSC sheets wrapped in tubes to produce a three-layer biomimetic urethra and labeled them with ultrasmall superparamagnetic iron oxide (USPIO) (Fig. 2). The grafts were subcutaneously transplanted for 3 weeks to promote vascularization and biomechanical strength of the biomimetic urethra, and the repair of dog urethral defects followed this. The graft maintains three layers of structures, including the epithelial, fibrous, and muscular layers. Three months after urethral reconstruction, the reconstructed urethra was functionally and morphologically comparable to the natural urethra. This three-layer epithelial fibromuscular tissue-engineered urethra can repair full-thickness urethral defects.

The advantage of cell sheet technology is that it removes the influence of scaffold material degradation. The cell sheet technique can construct a multilayer biomimetic urethra according to the anatomical characteristics of the urethra. The cell sheets retain close contact between cells and have natural intercellular tissue between cell connections, which is beneficial to maintain cell viability and promote cell growth and proliferation at the reconstructed site. However, the cell sheet cannot provide sufficient mechanical strength and spatial support [41]. The application of cell sheets reduced inflammation and fibrosis and eliminated biocompatibility problems, thereby improving the success rate of urethral reconstruction. A significant question that remains to be resolved is how to improve the graft’s mechanical strength.

## Scaffolded grafts

Many materials have been used to repair urethral defects, including acellular matrix, natural polymer, synthetic polymer, and composite scaffolds [42]. However, the structural design of the scaffold itself also plays a considerable role in urethral reconstruction. We divided the scaffolds used for urethral repair into core-shell and layered scaffolds at the microscopic and macroscopic levels of the structure. In layered scaffolds, those that can perform their functions macroscopically, including: providing 3D support, cell adhesion, or mechanical strength, are considered a single layer. And it is classified according to the macroscopic layers of the scaffolds. The invention of core-shell and layered scaffolds provides a new strategy for urethral reconstruction, which has a gradient structure and unique biomechanical properties. Table 2 summarizes the scaffold grafts cited in this article for urethral reconstruction.

**Table 2:** Overview of scaffold grafts in urethral reconstruction

Scaffold grafts	Biomaterials	Animals for modeling	Repaired length	Follow-up/study period	In vivo/in vitro	Results	References
Core-shell structure scaffolds	Collagen/P(LLA-CL)	rabbit	2 cm	3 months	In vivo	wide caliber; less collagen; more smooth muscle; thicker epithelium	[58]
	Collagen/P(LLA-CL)	dog	2 cm	12 weeks	In vivo	abundant ECM; better plasticity and strength; Inhibit fibrin deposition	[59]
Single-layer scaffolds	BAM	rabbit	2 cm	6months	In vivo	multiple layers of continuous urethral epithelium; upregulated the expression of myosin	[61]
	BAM	canine	6 cm	12months	In vivo	continuous muscle layers and epithelial layers; wide caliber	[62]
	Porcine urethras	NA	NA	14days	In vitro	Imitate the microstructure and composition of natural tissues	[63]
	BC	Rabbit	2 cm	3months	In vivo	Improve cell biological activity; Low immunogenicity	[66]
	BC/BAM	Rabbit	1 cm	3months	In vivo	Accelerate angiogenesis; Wide caliber; Multiple layers of continuous urethral epithelium	[67]
	SF	Canine	5 cm	6months	In vivo	Complete epidermal layer and fibrolblast layer structure	[70]
	hdCGTs	rabbit	2 cm	3months	In vivo	Wide caliber; Improve graft stability; Low immunogenicity	[72]
	CCC	Minipig	2 cm	24 weeks	In vivo	Multiple layers of continuous urethral epithelium; Low immunogenicity; Outstanding stability and storability	[74]
	cPUU	rabbit	1.5 cm	3 months	In vivo	High mechanical strength; Wide caliber; Reduced complication rate	[75]
	Human amniotic	canine	3 cm	8 weeks	In vivo	Promote angiogenesis; Multiple layers of continuous urethral epithelium; Less scar tissue	[78]
	AM	rabbit	1 cm	3months	In vivo	Simplify surgery procedure; Reduce postoperative complications; Good biocompatibility and low immunogenicity	[80]
	AM	rabbit	2 cm	1months	In vivo	Low immunogenicity; High blood vessel density	[92]
Bilayer composite scaffolds	Epithelial-muscular	Canine	1 cm	3months	In vivo	Effectively mimick native structure of urethra	[82]
	BSM- Autologous urethral tissue	Rabbit	2 cm	12 weeks	In vivo	Avoid cell expansion procedures; Excellent processability; Nonimmunogenicity	[83]
	SF-SF	Rabbit	2 cm	3months	In vivo	High porosity; Help smooth muscle and epithelial tissue regeneration	[84]
	SF-SF	Rabbit	1 cm	3months	In vivo	Neurovascularized urethral tissue; Wide caliber	[87]
	BAMH/SF	Rabbit	2.5 cm	3months	In vivo	Promote vascularization; Promote regeneration of urothelium and smooth muscle; Wide caliber	[89]
	BAMG/SF	rabbit	1.5 cm	3months	In vivo	Enhance cell adhesion and proliferation; Reduce collagen deposition; Promote vascularization	[91]
	BC-SF	canine	5 cm	3months	In vivo	High porosity; Facilitate cell adhesion and proliferation; Promote angiogenesis	[93]
	PLLA/PLGA/PCL-PLLA/PLGA/PLCL	Rabbit	2 cm	6months	In vivo	layered repair of urethra; wide caliber	[95]
	Collagen/ Elastin	NA	NA	NA	In vitro	Mimic natural structure; Excellent mechanical properties; Promote cell infiltration	[96]
Tri-layer composite scaffolds	PLA-PHBV-PLA	NA	NA	28days	In vitro	None-immunogenicity; High mechanical strength	[97]
	PLLA/ Gelatin	Rabbit	2.2 cm	3months	In vivo	Oriented SMC; Wide caliber; Promote angiogenesis	[102]
	Collagen-PCL-collagen	NA	NA	14days	In vitro	Facilitate cell adhesion and proliferation; High mechanical strength	[103]
	PU-alt copolymer	Rabbit	2.2 cm	3months	In vivo	Promote the expression of α-SMA and AE1/AE3; Induce immune cell apoptosis; Simulate urethral structure	[107]
	PU-alt copolymer	canine	2.2 cm	3months	In vivo	Promote vascularization; Mimic natural structure; Good mechanical properties	[109]
	PCL/PLCL	NA	NA	NA	In vitro	High porosity; High mechanical strength	[111]
Other multilayer scaffolds	SIS	Rabbit	1 cm	9months	In vivo	Complete urothelium layers; No effective aggregation of smooth muscle cells	[112]
	SIS	NA	Average 2.7 cm	Average 23months	In vivo	Insufficient mechanical strength and degradation; Affect cell adhesion and proliferation	[113]

### Core-shell structure scaffolds

In this review, we defined the core-shell structure scaffold as a tissue engineering scaffold made of core-shell fibers. This is based on the microscopic level of the scaffold structure. The core-shell fiber structure has two separate parts: the inner part (‘core’) and the outer part (‘shell’). Since they are separated in space, each core and shell can perform independent functions. However, they all have interfaces, and the molecules are permeable so that molecular interactions can occur between them. The role of the core, which is thought to be loaded with biological factors, is to deliver or fix tissue cells and provide them with 3D culture environments. The shell protects the internal physical components, controls the release of core molecules, and protects living cells [43]. The core-shell fiber has a large specific surface area, achieving a high concentration of bioactive surface groups and enhancing cell adhesion. By selecting the total fiber diameter and deposition method, the scaffold’s total porosity and pore size were controlled to achieve cell diffusion and ingrowth [44].

The preparation methods of core-shell scaffolds mainly rely on electrospinning-based approaches, including coaxial, emulsion, and single electrospinning. Furthermore, in situ posttreatments such as metal sputtering, electrochemical deposition [45], and UV photocrosslinking [46], as well as reoxidation with thermal pretreatment [47, 48], are used. Coaxial electrospinning is the most widely used manufacturing technology for core-shell scaffolds [49]. Compared with other types of scaffolds, core-shell scaffolds demonstrated unique advantages. The core-shell scaffold provided a 3D microenvironment for cell attachment, proliferation, and differentiation. In addition, core-shell fibers can enhance the mechanical properties of natural polymers and maintain good biocompatibility by incorporating polymers into the core layer of the scaffolds. Blackstone et al. [50] incorporated poly-L-lactic acid (PLA) and poly(ε-caprolactone) (PCL) core layers into gelatin to make a core-shell scaffold. The core-shell scaffold demonstrated higher mechanical properties than single-layer gelatin scaffolds. Xu et al. [51] fabricated a core-shell fiber consisting of silk fibroin/poly(L-lactic acid-co-ε-caprolactone)-polyethylene oxide (SF/PLCL-PEO), which could continuously release connective tissue growth factor (CTGF) and fibroblast growth factor 2 (FGF-2). The sustained release of growth factors improved the proliferation and fibrogenesis of mesenchymal stem cells (MSCs), promoting the regeneration of connective tissue of the urethra.

**Fig. 3:**
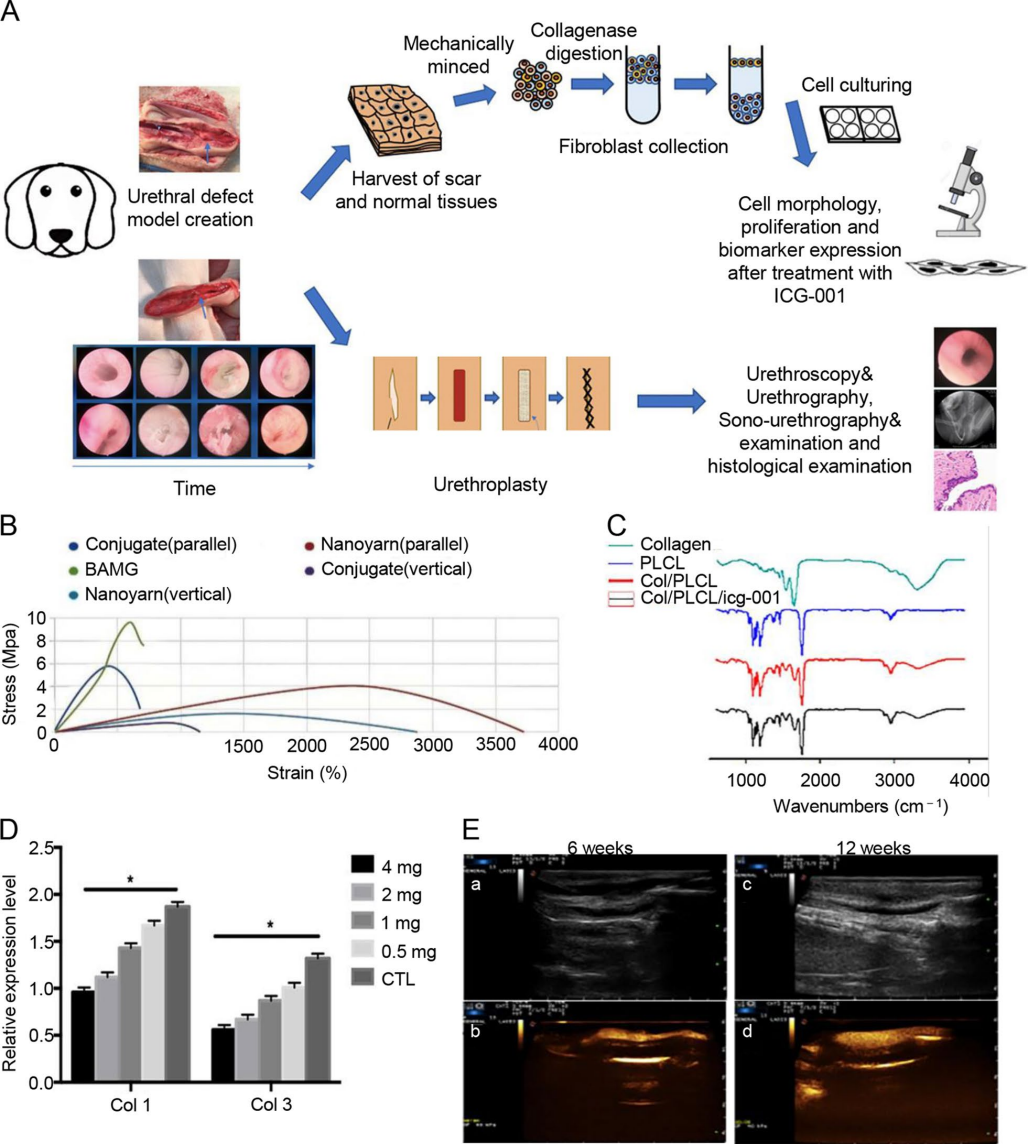
Overview of key components in the study. **A** The flowchart of study design. **B** The tensile strength of nanoyarn, conjugated scaffold and BAMG. **C** FTIR for different polymers in PLCL, control nanoyarn and drug (ICG-001) delivering nanoyarn. **D** Relative expression quantification of fibrosis related proteins [Collagen type I (Col I), Collagen type III (Col III)] were evaluated. *indicates significant difference (*p <* 0.05, comparing to control). **E** Sonourethrography and ultrasonic contrast examinations for the urethras repaired with ICG-001-delivering nanoyarn. **a**, **c** Sonourethrography examination results. **b**, **d** Ultrasonic contrast examination results

Core-shell scaffolds can avoid direct contact between biomolecules and organic solvents and protect active molecules in the microenvironment. Since the core-shell scaffold has many advantages, it has been applied to the regeneration and reconstruction of bone [52, 53], nerves [54, 55], blood vessels [56], and myocardium [57]. The core-shell scaffold also has excellent application prospects in urethral tissue engineering. Zhang et al. [58] fabricated a collagen/poly(L-lactide-*co*-caprolactone) (P(LLA-CL)) scaffold loaded with a Wnt pathway inhibitor (ICG-001) by coaxial electrospinning. The scaffold highly imitated the ECM in structure and morphology and demonstrated good mechanical properties. The core-shell structure allows the continuous delivery of ICG to significantly inhibit ECM deposition and fibrosis production in fibroblasts. Compared with the nondrug scaffold, the scaffold loaded with the Wnt pathway inhibitor promoted the regenerated urethra with more smooth muscle and multilayered urothelium but less collagen. After that, the group improved the structure of the scaffold and developed a core-shell collagen/P(LLA-CL) nanoyarn-based scaffold, which was used to repair urethral defects in dogs (Fig. 3) [59]. Some researchers have used nanoyarn/hydrogel core-shell scaffolds to mimic natural skeletal muscle tissue [46]. Compared with traditional conjugated nanofiber scaffolds, nanoyarns have been proven to increase the pore size [60]. The nanoyarn has better plasticity and strength and is suitable for operation and suture during surgery. Three months after implantation in dogs, it was found that the ICG-001-delivering nanoyarn-based scaffold can better deliver ICG-001 continuously, thereby more effectively inhibiting fibroblast proliferation and fibrotic protein expression [59].

### Single-layer scaffolds

Single-layer scaffolds are a typical scaffold structure in urethral tissue engineering and have been widely studied in preclinical and clinical trials. Such scaffolds are often planted with somatic or stem cells based on a single layer of material, and the spatial distribution of different types of cells is not very clear. Using decellularized technology to prepare decellularized biological scaffolds to repair urethral defects is a relatively common strategy. Li et al. [61] seeded epithelial-differentiated rabbit adipose-derived stem cells (Epith-rASCs) into bladder acellular matrix (BAM) scaffolds to treat a 2-cm urethral defect in male rabbits. After implantation, multiple layers of continuous urethral epithelium showed good urethral continuity after 6 months. The expression of myosin was upregulated in neourethral tissue, which was beneficial to repair muscle bundles in urethral tissue and prevent urethral stricture caused by lumen contracture. Early formation of urothelium has been reported to avoid the recurrence of inflammation and stenosis caused by urine erosion. Orabi et al. [62] seeded bladder epithelial cells and SMCs in BAM scaffolds and implanted the scaffold into long urethral (6 cm) defects in canine preclinical models. Complete continuous muscular and epithelial layers were found after 1 month of urethroplasty, and all reconstructed urethras showed a wide caliber without stricture after 12 months. Simões et al. [63] decellularized porcine urethra to produce acellular scaffolds. The scaffolds retained the microstructure and biochemical composition of natural tissues and were suitable for cell adhesion, proliferation, and myofiber formation. To investigate the myogenic differentiation of cells in the scaffold, human muscle progenitor cells (MPCs) were cultured on the urethral smooth muscle and skeletal muscle-derived matrix. MPCs are differentiated into different phenotypes in a different matrix. This study demonstrated that tissue-specific ECM could regulate cell behavior and affect cell differentiation.

**Fig. 4:**
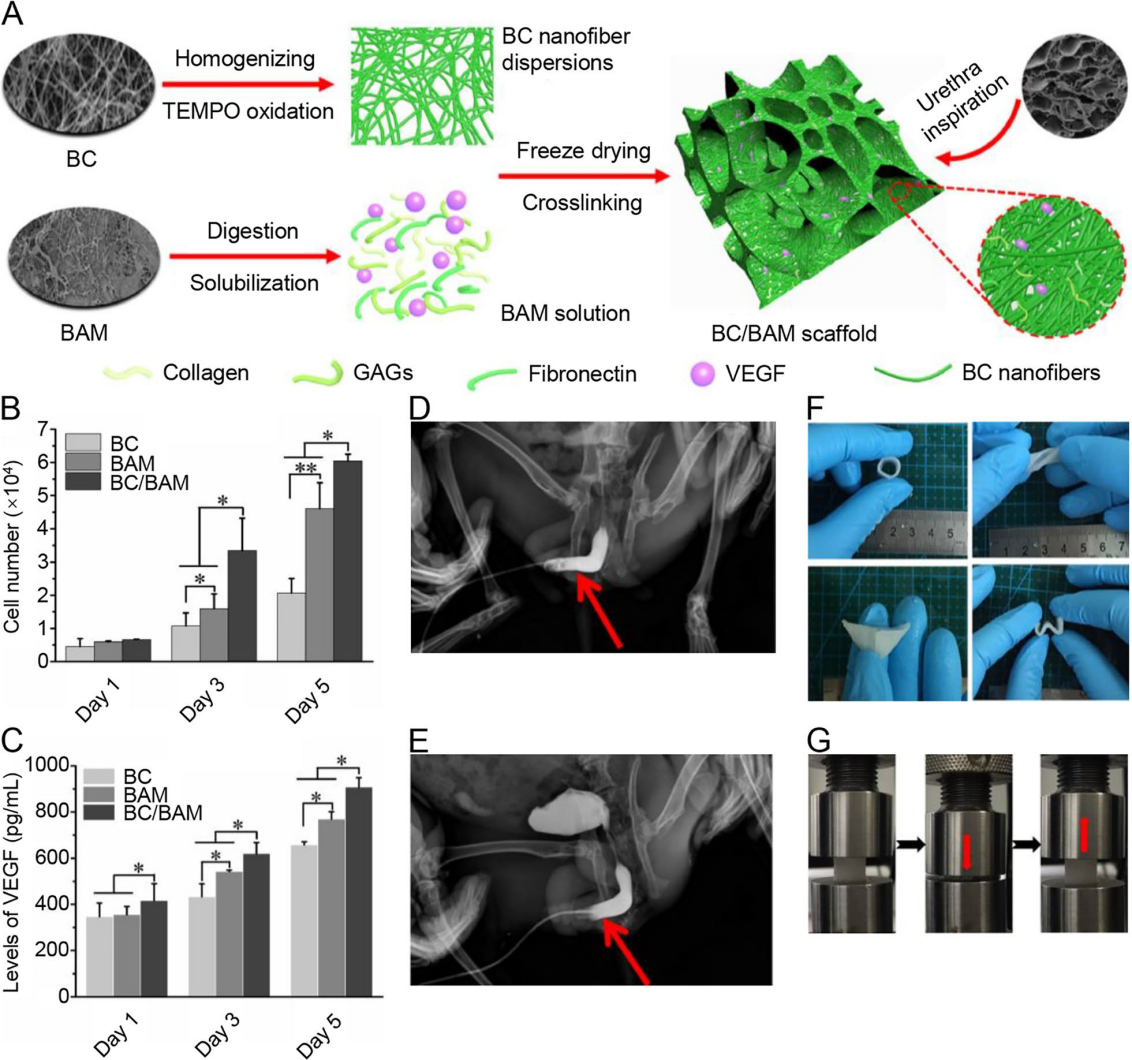
Design, processing and architecture of the bioinspired BC/BAM scaffold. **A** Schematic showing the preparation of BC/BAM scaffold. **B** Cell number of HUVECs cultured on BC, BAM and BC/BAM scaffolds measured by CCK-8 assay. **C** ELISA detection for levels of VEGF in BC, BAM and BC/BAM group during in vitro culture. Urethrography images of BC/BAM (**D**, **E**) group. The red arrows represent the urethrography site of the urethra. **F** Photographs for the shape adaptability of BC/BAM scaffold. **G** Photographs of the BC0.5/BAM0.5 scaffold under a compressing and releasing cycle

Bacterial cellulose (BC) derived from *Acetobacter xylinum has* become an effective biomedical scaffold because of its high mechanical strength, satisfactory biocompatibility, and unique nanostructure [64, 65]. Huang et al. [66] utilized a gelatin sponge to interfere with BC fermentation and successfully created 3D porous BC scaffolds. Rabbit lingual keratinocytes were seeded into 3D porous BC scaffolds to repair 2-cm urethral defects in the rabbit model. This 3D porous BC scaffold imitated the structural characteristics of the ECM and improved the biological activity of seeded cells. In addition, the scaffold effectively enhanced the regeneration of urethral tissue without causing inflammation or stricture. Inspired by the natural urethra, Wang et al. [67] believe that constructs that mimic the natural urethra to the utmost extent may effectively promote urethral reconstruction. Therefore, they developed a 3D porous nanofiber structure scaffold using BC and dissolved BAM (Fig. 4). The dissolved BAM still retains ECM components, including collagen and growth factors, while BC nanofibers (30–100 nm) can mimic the nanomorphology of the ECM. Vascular endothelial growth factor (VEGF) is incorporated into scaffolds to promote blood vessel formation. In vitro studies have shown that biomimetic BC/BAM scaffolds promote angiogenesis by promoting the growth of human umbilical vein endothelial cells and the expression of proteins related to endothelial function. Simulating the spatial structure of ECM and VEGF promoted the formation of blood vessels and epithelium and accelerated the regeneration of the urethra, and the 1-cm rabbit urethral defects were successfully repaired.

The excellent biocompatibility and low immunogenicity made silk fibroin (SF) and high-density collagen gel tubes (hdCGTs) become outstanding biomaterials for use in tissue engineering [68, 69]. Xie et al. [70] chose electrospinning SF matrices stretched in ethanol. This processing increased the fracture strength of natural SF by five times [71]. Autologous keratinocytes and fibroblasts were seeded into the stretched SF matrix to repair the 5-cm long canine urethral mucosal defects. Both cell lines exhibited proliferative capacity in the scaffold, forming a complete multilayered epidermal and fibroblast layer 6 months after surgery. Micol et al. [72] evaluated high-density collagen gel tubes as urethral grafts to repair rabbit urethral defects. The histology of cellular and autologous smooth muscle cell-seeded hdCGTs did not show any signs of inflammation. Spontaneous regeneration of the urothelium was found in all grafts. The caliber of the neourethras (96.6% of the normal caliber) repaired by the hdCGTs seeded with cells was closer to that of the natural urethra. Single-layer collagen matrix grafts can promote the viability and proliferation of urothelial cells and can improve the stability of cell-based implants [73]. Sievert et al. [74] seeded high-density urothelial cells into a collagen type I-based cell carrier (CCC) for penile urethroplasty. After induction and culture, CCCs formed stratified multilayer autologous urothelium. The seeded single-layer scaffold shows excellent suitability and stability after manipulation and application. Six months after being implanted in the minipigs, the seeded CCC was well integrated into the host tissues, and there was no sign of inflammation, recurrence of stenosis, or rejection.

The amniotic membrane (AM) is an excellent biocompatible material with great potential for urethral reconstruction [76]. AM comprises three essential structures: the epithelium, basement membrane, and vascular stroma layer. AM has excellent low immunogenicity and scar reduction properties and promotes the migration and proliferation of epithelial cells [77]. Chen et al. [78] implanted allogeneic bone marrow stromal cells (BMSCs) and endothelial progenitor cells (EPCs) into a decellularized human amniotic scaffold (dHAS) to repair long segment circumferential urethral defects. BMSCs mainly differentiate into urothelial cells, while EPCs mainly differentiate into vascular endothelial cells. These cells jointly participate in the repair of the urethra. Two months after the operation, an examination showed that multiple layers of the complete urothelium covered the neourethra, and the submucosa had abundant blood vessels [79]. Wang et al. [80] separated the basement layer of AM to obtain a denuded dHAS, and then rabbit urothelial cells were seeded on the surface of the dHAS scaffold to repair rabbit urethral injury. Tissue-engineered dHAS is easy to perform in urethroplasty, reducing the operation procedure time and the risk of postoperative complications. The scaffold exhibits a tissue structure similar to urethral tissue and can be used as an ideal urological reconstruction implant with low immunogenicity.

The preparation of single-layer urethral scaffolds is relatively easy, and the spatial structure of the materials used simulates natural ECM. However, single-layer scaffolds cannot effectively distribute different cells because they are different from the natural urethral structure. Therefore, this method is too difficult to treat long and complicated urethral defects.

### Bilayer composite scaffolds

**Fig. 5:**
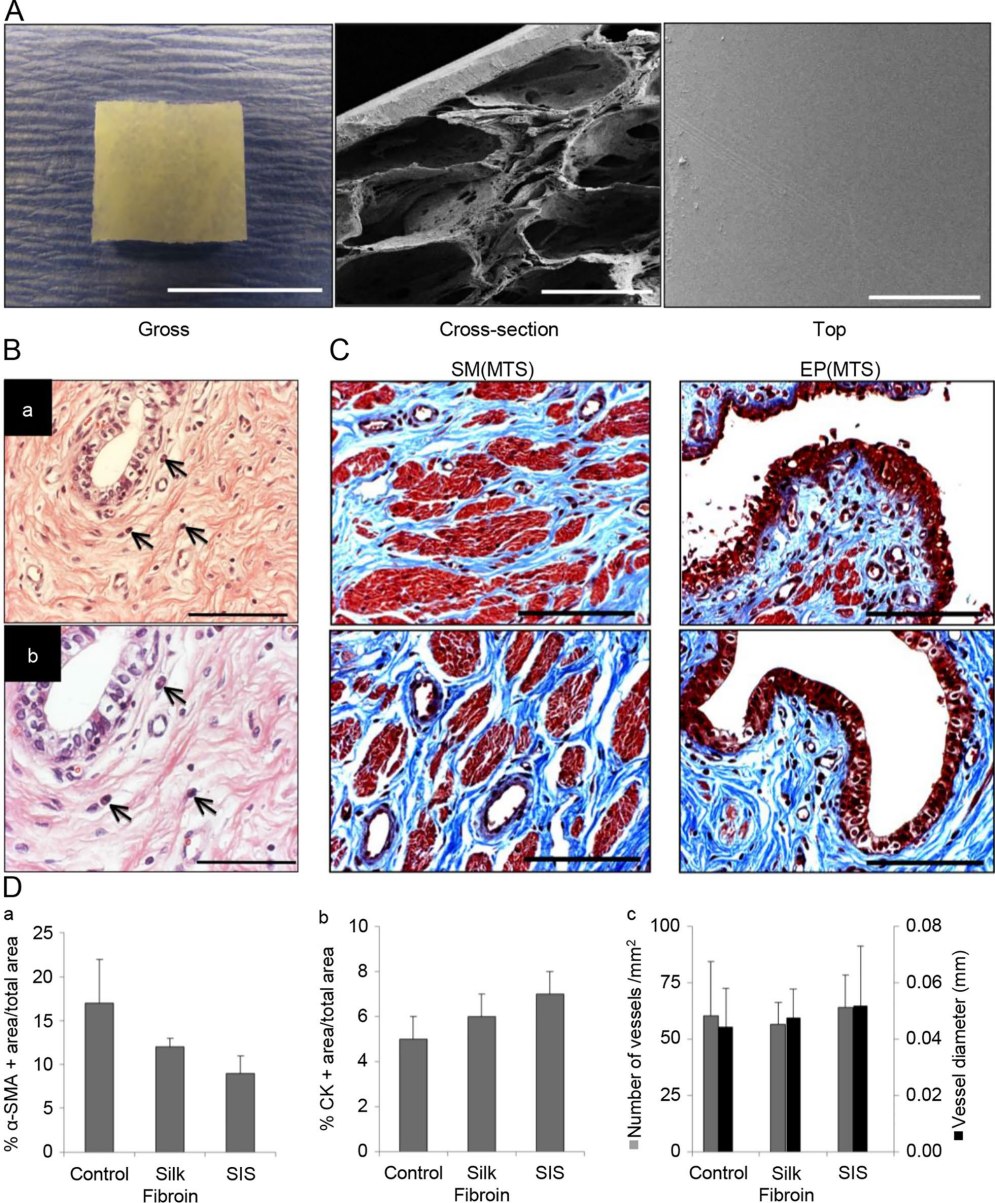
Overview of key components in the study. **A** Structural characterization of silk fibroin scaffold. Photomicrographs of gross scaffold morphology (scale bar: 1 cm) and SEM images of cross-sectional and top views of bilayer scaffold architecture (scale bars: 400 mm). **B** Eosinophil granulocytes (acute inflammation, denoted by arrows) present in de novo tissue supported by silk fibroin scaffolds. **a** Scale bars: 200 mm, **b** scale bar: 100 mm. **C** Histological evaluations (MTS analyses) of urethral tissue regeneration in control and implant groups following 3 m postop. Magnified de novo smooth muscle (SM) and epithelial (EP) tissue formation displayed in 3rd column. Scale bars: 200 mm. **D** Histomorphometric analysis of the extent of regenerated a-SMA + smooth muscle bundles (**a**), CK + epithelium (**b**), and CD31 + vessels (**c**) present in the original surgical sites of control and scaffold groups

The urethra comprises the urothelial and smooth muscle cell layers as an excretory organ. In the strategy of urethral reconstruction, some researchers designed double-layer structure scaffolds according to the structural characteristics of the urethra. They seeded different cell types onto the double-layer scaffolds to allow tissues to grow in layers. Mechanical stimulation has been found to effectively induce ADSCs to differentiate into functional smooth muscle tissue [81]. Fu et al. [82] seeded mechanically-induced ADSCs and oral mucosal epithelial cells on a biodegradable polyglycolic acid (PGA) tubular scaffold using a layered seeding technique. The bioreactor was then used to construct a tissue-engineered epithelial-muscular urethra with a bilayer structure. The scaffold consists of an epithelial cell lining and an outer muscle layer, which effectively simulates the structure of the urethra and successfully repairs the canine urethra. Chun et al. [83] evaluated the efficacy of a bilayer scaffold consisting of acellular bladder submucosa matrix (BSM) and autologous urethral tissue to treat long urethral strictures. To avoid the cell expansion procedure in vitro, BSM was combined with autologous tissue to repair urethral defects. The incorporation of graft and host tissue improved the integration of neotissue with the surrounding tissue. BSM autologous tissue grafts showed excellent processability and nonimmunogenicity, successfully promoting the regeneration of urethral defects. Chung et al. [84] compared the ability of bilayer SF scaffolds and small intestinal submucosa (SIS) to repair urethral defects in rabbits (Fig. 5). The results showed that the bilayer SF scaffold promoted angiogenesis and neurogenesis in smooth muscle and epithelial tissue during urethral reconstruction. The high porosity of SF allowed surrounding tissue to grow into the scaffold, and the annealed SF membrane could prevent erosion from urine [85, 86]. Porous SF mimics the mucosal layer of the urethra, and dense SF membranes mimic the epithelial layer of the urethra. Moreover, SIS caused chronic inflammation, while the bilayer SF scaffold seldom caused inflammation in the repaired area.

Bladder acellular matrix usually exhibits weak adjustability during the decellularization process, while hydrogels are used in tissue engineering because of their variable geometry, adjustable mechanical strength, and porous structure [88]. Cao et al. [89] developed porous BAM hydrogels (BAMHs) by pepsin-enzymatic dissolution. Similar to BAM, BAMH can also preserve endogenous growth factors, including VEGF and keratinocyte growth factor (KGF) [90]. They prepared prevascularized BAMH/SF composite scaffolds to repair rabbit urethral defects [89]. Histological analysis showed that the prevascularized BAMH/SF composite scaffold exhibited intact multilayered urothelium 1 month after implantation. The neourethra’s smooth muscle content and blood vessel density increased significantly at 3 months. Liu et al. [91] incorporated stromal cell-derived factor-1α (SDF-1α) into aligned SF and developed an SDF-1α-SF/3D porous bladder acellular matrix graft (3D-BAMG) composite scaffold. The SDF-1α-SF/3D-BAMG composite scaffold supports the migration of ADSCs and BMSCs, and SDF-1α can be released continuously in vivo and in vitro. The composite scaffold was implanted into the rabbit to repair the 1.5-cm urethral defect. Three months after the operation, the reconstructed urethra had sufficient patency and continuity. SDF-1α-aligned SF nanofibers can promote the regeneration of the urethral mucosa, smooth muscle, and capillary system, enhance cell adhesion and proliferation, and reduce collagen deposition.

Zhang et al. [92] combined rabbit skin epithelial cell sheets (SECs) with decellularized AMs for freezing treatment to reconstruct the rabbit urethra. The study found that the combination with decellularized AMs can reduce the cryopreservation damage of SECs. AMs can also enhance the mechanical strength of SECs. The cryopreservation method minimizes the infiltration of inflammatory cells in the graft and increases the blood vessel density. This bilayer urethral scaffold reduces immunogenicity and promotes vascularization through cryopreservation, showing good clinical application prospects.

Lv et al. [93] developed a BC-SF bilayer scaffold for urethral tissue engineering. The porous SF structure in the inner layer provided a microenvironment for cell adhesion and proliferation, while the dense cellulose in the outer layer offered a barrier to prevent the leakage of urine. BC-SF scaffolds significantly promoted angiogenesis in regenerated tissues. Wan et al. [94] developed a bilayer porous heterogeneous nanofiber scaffold using a similar layering principle. The bilayer nanoscaffold is divided into a microporous inner and a macroporous outer layer. The outer macroporous side is composed of PLLA/poly(lactic-co-glycolic acid) (PLGA)/PCL, and the microporous luminal side was prepared using PLLA/PLGA/PLCL. The loose outer layer provides enough 3D space for vascularization and smooth muscle growth, while the dense inner layer promotes the growth and reproduction of the urothelium. After that, the group seeded hypoxia-preconditioned ADSCs on this bilayer heterogeneous nanofiber scaffold to repair rabbit urethral defects. Hypoxic pretreatment of ADSCs can promote vascular regeneration and upregulate glycolysis, while the bilayer structure of the scaffold successfully supports the layered growth of epithelium and smooth muscle. With this, they successfully repaired a 2-cm long rabbit urethral defect [95]. Cunnane et al. [96] fully characterized the mechanical characteristics and structural components of the human urethrae and found that the urethrae contain a large amount of elastin and collagen at a ratio of 0.3. Moreover, elastin and collagen significantly affect tissue mechanics. Elastin plays a more significant role in low intracavitary pressure, and collagen regulates stiffness and incremental modulus at medium and high pressures. Using the composition and structural characteristics of the urethra, they designed a bilayer scaffold using elastin and collagen. The bilayer scaffold comprises an internally dried dense film layer and an external porous freeze-dried layer. Compared with the current gold standard tissue engineering material, SIS, it is found that its cell infiltration is significant, and its mechanical properties, composition, and structure are closer to those of the normal human urethra.

### Tri-layer composite scaffolds

Using concepts of designing urethral bionic scaffolds based on the structural characteristics of the urethral tissue, the design of three-layer composite scaffolds (similar to bilayer composite scaffolds) is a choice for some scholars. While designing the trilayer scaffold structure, one needs to rationally plan the seeding of different cells to regenerate the tissue structure in layers. The increase in the number of layers makes it possible to fully allocate and utilize the functions of each layer. Some are mainly responsible for cell infiltration and growth, and some enhance the scaffold’s mechanical strength and spatial support. Therefore, a trilayer bionic scaffold may be a suitable choice from the structural point of view.

Simsek et al. [97] used microfibrous PLA and nanofibrous poly(3-hydroxybutyrate-co-3-hydroxyvalerate) (PHBV) to prepare PLA-PHBV-PLA micro/nanofiber trilayer scaffolds for urethral reconstruction. PLA is a well-known degradable biomaterial with good biocompatibility that has been widely used in tissue engineering [98–100]. PHVB can be easily spun into nanofibers, and the combination of PLA microfibers with PHVB developed a microporous/nanoporous/microporous trilayer structure that imitated the dermis structure [101]. The PHVB nanofiber layer acted as a barrier for cell penetration, while the PLA microfiber layer promoted the adhesion and proliferation of two different cell types. Compared with decellularized biological scaffolds, micro/nanofibrous tri-layer scaffolds avoided immune rejection and viral infection. Liu et al. [102] designed a flexible poly(L-lactic acid) (PLLA)/gelatin tubular nanofiber scaffold (75:25) with hierarchical architecture. They seeded ECs into the inner layer and SMCs into the middle and outer layers. The PLLA/gelatin nanofiber scaffold (75:25) is highly hydrophilic and simultaneously significantly promotes the adhesion and proliferation of ECs and SMCs. The PLLA/gelatin trilayer scaffold (75:25) also upregulated the expression of actin (α-SMA) in SMCs and keratin (AE1/AE3) in ECs. Three months after implantation in the rabbits, the neourethra remained unobstructed and promoted the remodeling of oriented SMCs and the formation of blood vessels and urothelium. Zhao et al. [103] designed a trilayer collagen-PCL-collagen scaffold for urethral repair. Because the mechanical strength of collagen is relatively low, Zhao plastically compressed collagen and extracted water to make a flat collagen sheet [104, 105]. PCL was responsible for mechanical support and elasticity transmission in the collagen-PCL composite scaffold, while collagen was beneficial for cell adhesion, proliferation, and differentiation. The incorporation of PCL and collagen sheets improved the mechanical properties of the scaffold and met the mechanical requirements in urethral tissue engineering. Excellent mechanical properties and biocompatibility made PCL-collagen scaffolds a promising method for urethral reconstruction.

Alternating block polyurethane (PU-alt) is a biodegradable material with highly controllable physical and chemical properties [106]. PU-alt synthesis technology regularly introduces biological materials with independent properties into its linear block to meet specific biological needs. Niu et al. [107] obtained a PU-alt nanofiber by adjusting the hydrophilic PEG and hydrophobic PCL components in the linear block of PU-alt. Nanofibers can simultaneously promote EC and SMC adhesion and proliferation and upregulate elastin synthesis. They used this nanofiber to design a tubular PU-alt scaffold with a layered structure. They designed the scaffold into three layers: inner, middle, and outer films. SMCs are uniformly seeded on each nanofiber membrane, and ECs are seeded on the internal films. This design of cell distribution improves the 3D distribution of planted cells and is closer to the physical interface of the natural urethra. Three months after the three-layer PU-alt nanofiber scaffolds were implanted in the rabbits, the 2.2-cm anterior urethral defects were successfully repaired. Recent studies have found that hydrophilic modification can improve the biocompatibility of scaffolds [108]. The PU-alt nanofiber scaffold is an amphiphilic structure composed of alternate hydrophilic PEG and hydrophobic PCL segments. Niu et al. [109] successfully repaired a 2.2-cm dog urethral defect using the PU-alt nanoscaffold. They found that such PU-alt nanofibers can upregulate the expression of elastin in SMCs and AE1/AE3 in ECs and transiently induce cytokine and chemokine responses to promote the recruitment of host inflammatory cells and the formation of new blood vessels. This indicates that based on a reasonable scaffold structure, stimulating cell proliferation through the material itself may be a promising direction for tissue engineering in the future.

**Fig. 6:**
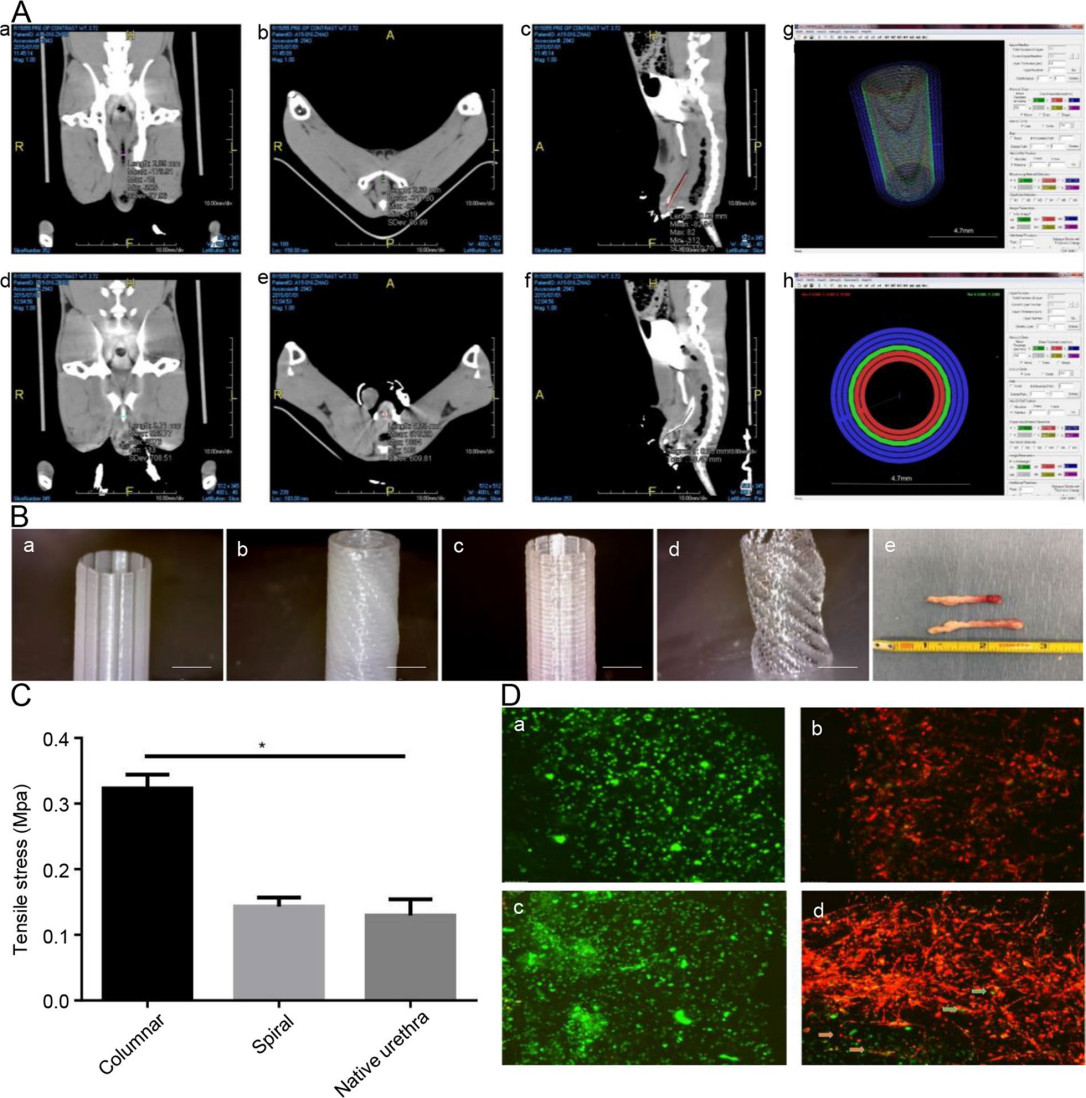
Design, processing and architecture of the PCL/PLCL scaffold. **A** (**a**, **b**) The CT scanning images of the urethra; (**d**-**f**) The CT scanning images of the urethra filled with contrast reagent; (**g**) 2D slice (single layer) of the urethra design made using the WFIRM printing code program; where Red: UCs-laden hydrogel; Green: scaffold made of PCL/PLCL 50:50 blend; and Blue: SMCs-laden hydrogel. (**h**) The 3D rendering of the urethral design with porous scaffold and two hydrogel layers as seen using the WFIRM printing code program. **B** (**a**) PCL scaffold with columnar design; (**b**) PCL scaffold with spiral design; (**c**) PCL/PLCL (50:50) scaffold with columnar design; (**d**) PCL/PLCL (50:50) scaffold with spiral design; (**e**) native rabbit urethra. Scale bar: 2 mm (**C**) Stress testing of spiral and columnar scaffolds with PCL/PLCL blend (50:50). (**D**) UCs (labeled with green fluorescent dye) as seen in the hydrogel component of the bioprinted urethral construct after 1 day and 7 days of culture (**a**, **c**) and SMCs (labeled with red fluorescent dye) in the hydrogel component of the bioprinted urethral construct after 1 day of culture (**b**, **d**). Scale bar: 100 μm

Three-dimensional bioprinting simultaneously processes multiple biological materials and cell types and provides a promising method for manufacturing complex tissues and organs [110]. The most significant advantage of 3D bioprinting is customizing organs using images and histological data according to clinical demands. Zhang et al. [111] developed an integrated bioprinting technology for the fabrication of spiral urethral scaffolds consisting of poly(ε-caprolactone) (PCL) and poly(lactide-co-caprolactone) (PLCL). UCs and SMCs were delivered to the inner and outer layers of the scaffold, respectively (Fig. 6). The 3D spiral scaffold demonstrated high porosity and mechanical strength equivalent to native rabbit urethra. The inner and outer layers of the hydrogel with embedded cells provide an environment for cell growth. After cultivation for 7 days, the spiral scaffold maintained the viability of UCs and SMCs, and the microenvironment of the scaffolds was beneficial to cell proliferation and urethral regeneration. Although the scaffold has only been tested in vitro, the combination of polymer and hydrogel coincides with the natural urethral anatomy.

### Other multilayer scaffolds

As early as 2003, Nuininga et al. [112] tried to use a four-layer SIS to repair the rabbit urethral stricture. The results of their study showed that complete urothelium layers could be observed 3 months after transplantation. However, no effective aggregation of SMCs was observed until the ninth month. They speculated that the aggregation of SMCs depends on the porosity of bioscaffolds. Orabi et al. [113] used four-layer SIS grafts to treat patients with hypospadias, and 9 of 12 patients were cured successfully. Four-layer SIS grafts demonstrated better mechanical properties and biocompatibility compared with single-layer SIS. Moreover, a posttransplant infection can be prevented by incorporating bacterial interfering agents and antimicrobials into the SIS; therefore, multilayer SIS grafts may be more suitable for urethral reconstruction than single-layer SIS grafts. Despite this, the performance of the four-layer SIS still cannot meet clinical requirements. The singleness of material types limits the mechanical properties and biodegradability of the scaffold, which affects the adhesion and growth of cells. This shows that multilayered scaffolds are beneficial to urethral repair, but it does not mean that the more layers of scaffolds there are, the better. The structure and materials of the scaffold need to be reasonably designed based on conforming to the physiological structure of the natural urethra. The simple stacking of the material and the inconsistency of the scaffold structure with the natural anatomical structure of the urethra results in the failure of the reconstructed urethra to grow normally. Bionic urethral scaffolds need to simulate the structure of the natural urethra. After mastering the anatomy, and physical and chemical properties of the natural urethra, the structure of the scaffold is designed with appropriate materials and cells rather than a simple stack of single materials.

## Discussion

The repair of urethral injury remains a significant challenge in urology. Various methods have been used for urethral reconstruction, but there is still no optimal solution to meet clinical requirements. Oral mucosal grafts and skin flaps are the earliest grafts used for urethral reconstruction, and donor complications such as bleeding, hematoma, and nerve damage [114] and high recurrence rates of urethral strictures limit their application in clinical practice. The development of tissue engineering has made significant progress in exploring the most suitable methods for urethral reconstruction. Many efforts have been made to develop new grafts to treat complex urethral injuries. However, a suitable graft has not yet been developed. The acellular matrix cannot guarantee the complete removal of cellular components, leading to a severe immune response. The degradation products of synthetic polymers could affect the microenvironment of urethral tissue. Cell implantation techniques also failed to ensure that cells were evenly distributed into the graft, affecting repair outcomes.

Many previous studies have focused on the biomaterials and cell sources used in grafts. Nevertheless, we believe that the graft structure is also a factor that must be considered in urethral reconstruction, whether microscopic or macroscopic. The number of layers of the graft should be optimized to fit the natural anatomy of the urethra. A reasonable multilayered structure of the graft can guarantee the uniform distribution of cells that is closer to the anatomical characteristics of the urethra. The multilayer design of the scaffolds allows clear division of labor for each part of the scaffolds. The microcontrol of the graft, including the structure and arrangement of fibers, can optimize the 3D microstructure, function, and mechanical strength of the construct. The scaffold structure should conform to the natural anatomy of tissues and organs in a macroscopic view. This is conducive to promoting the accumulation of surrounding autologous tissue cells to the reconstruction site, optimizing the structural composition of the new tissue, and increasing the survival rate of the graft. The graft conforming to the natural anatomical structure can effectively shorten tissue repair time, improve reconstruction efficiency and success rate, and better meet clinical needs. This strategy applies to all areas of tissue engineering. Wang et al. [115] developed a hydrogel/nanoyarn core-shell scaffold for nerve tissue engineering. The hierarchically aligned core-shell scaffold mimics the structure of naturally arranged nerve fibers, and the hydrogel shell mimics the epineurium layer that protects nerve cell tissue in the natural environment. By mimicking the hierarchical arrangement of natural nerves, this scaffold successfully induces neurite extension and arrangement. In contrast, core-shell nanofibers and nanoparticles may be an alternative direction at the microstructural level. Core-shell scaffolds have been widely used in drug delivery because of their particular properties [116]. They also have excellent application prospects in urethral reconstruction. The specific structure of core-shell nanofibers makes them have functions that many materials cannot perform. The shell part ensures sufficient biological strength and provides a surface for cell adhesion, while the core part can be loaded with growth factors or living cells [117]. The unique structure of core-shell nanofibers enables the scaffold to continuously release growth factors, thereby maintaining cell viability and proliferation. Such a scaffold can provide a 3D microenvironment that supports cell growth. Because of the excellent performance and various preparation methods, core-shell nanoparticles may also be a good choice for urethral reconstruction [118]. The functional characteristics of core-shell scaffolds are very suitable for urethral reconstruction. We believe that the combination of microscopic core-shell structures and macroscopic layered structures may be a promising development direction for urethral repair. However, the appropriate components of the scaffolds and cell sources still need to be investigated.

## Future perspectives

Compared with other areas of tissue engineering, urethral tissue engineering is still in its infancy. The discovery of new materials and methods can further develop urethral tissue engineering. Novel materials, preparation methods, and combinations can provide new inspiration for constructing urethral grafts, optimizing the graft structure from the micro- and macro-perspective. Mandal et al. [119] repaired long urethral strictures using tissue-engineered bovine pericardium. In a study of urethral reconstruction by partial bovine pericardial reconstruction, most patients successfully reconstructed urethral tissue during the 8 months of follow-up. This study successfully applied cardiovascular tissue engineering products to urology and achieved good results in urethral reconstruction.

A reasonable support structure requires advanced manufacturing methods to perform optimally. 3D bioprinting has been applied to urology because of its precise material structure control and high flexibility [120–122]. Computers control 3D bioprinting for additive manufacturing, so the structure of the biological constructs can be precisely controlled. Printing technology can process multiple cells and biomaterials simultaneously in a single step to produce grafts with complex structures [123]. This ability to highly control the 3D structure of biological constructs is necessary for urethral tissue engineering. Many interesting printing methods have been developed, which will produce a variety of interesting structures. These may be used for reference to urethral tissue engineering. Robu et al. [124] used multicell spheres as sacrificial materials and performed 3D printing on the background of the cell-laden hydrogel. Sacrificial cell spheres can be used for bioprinting perfusable tissue structures and printing hollow structures with finer and more complex structures. Ozbolat et al. [125] developed a microfluidic channel manufacturing method that can directly bioprint cell microfluidic channels in the form of hollow tubes. Most cells remained viable in the microfluidic channel wall. Using microfluidic printing technology, Pi et al. [126] successfully used human urothelial cells and outer human bladder SMCs to fabricate tubular urothelial tissues. These bioprinted tubular tissues can be actively perfused with nutrients to promote the proliferation of cells in different layers of the hollow structure. Kessel et al. [127] used physical pressure to pass the precrosslinked hydrogel through a screen to form microstrands. Such microstrands can form an entangled porous structure, which is stable in aqueous media. The entangled microstrands have excellent rheological properties and anisotropy after extrusion. Cells can be cultivated both inside and outside the hydrogel microstrands, which are compatible with the layered reconstruction of the urethra.

The ultimate goal of tissue engineering is to effectively apply the products in the clinic. To achieve this goal, effective cell extraction and scaffold preparation are essential. Urine-derived and adipose-derived stem cells are easy to extract and have large reserves, which are promising options for urethral tissue engineering [128, 129]. However, more important is the rapid and accurate preparation of the scaffold. In clinical practice, the size of the scaffold needs to be individually designed according to the patient’s condition [130]. Three-dimensional bioprinting’s high degree of control over the space structure of the scaffold and efficient preparation allow it to have promising application prospects. The development of new hydrogels also provides more possibilities for urethral reconstruction [131]. Therefore, from the perspective of the structural design of the urethral scaffold, we believe that the combination of new hydrogels and 3D bioprinting may be a possible future direction for repairing the urethra and that 3D printing will be a powerful tool for tissue engineering in the future.

## Conclusion

We reviewed the various grafts used in urethral reconstruction and classified them according to their structure to determine a structure suitable for urethral repair. Although various structure types have been used for urethral reconstruction, developing a more reasonable structure is still challenging. Appropriate biomaterials and graft preparation techniques are the basis for these techniques. In conclusion, we believe that the combination of microscopic core-shell structures and macroscopic layered structures may be a promising development direction for urethral repair. Core-shell nanostructures, new hydrogels, and 3D bioprinting provide the possibility for the realization of this idea.

## Data Availability

Not applicable.
